# 

**Published:** 1833-04-01

**Authors:** 


### BIBLIOGRAPHICAL RECORD;

OR,

*Works received for Review since the last Quarter.*

1. A Treatise on the Diseases of the Liver and on Bilious Complaints; with Observations on the Management of the Health of those who have returned from Tropical Climates, and on the Diseases of Infancy. By GEO. HAMILTON BELL, late Residency Surgeon, Tanjore. Octavo, pp. 152. Dec. 1832.

2. The Dublin Journal of Medical and Chemical Science, &c. No. VI. January, 1833.

3. The Physician's Vade-Mecum; or a Manual of the Principles and Practice of Physic, &c. By ROBT. HOOPER, M.D. New Edition, considerably enlarged and improved, 1833.

☞ *Messrs. Renshaw and Rush have sent forth a new edition of Dr. Hooper's excellent Manual, under the superintendence of an erudite physician, who has much improved the original.*

4. Traité Pratique des Maladies de l'Uterus et ses Annexes, fondé sur un grand Nombre d'Observations Cliniques. Atlas de 41 Planches in folio, gravées et coloriées, représentant les principales Alterations Morbides des Organes Genitaux de la Femme. Par Mme. Veuve BOIVIN, et par A. DUGES, Professeur, &c. Atlas, quatrième Livraison, 1833. Bailliere, Regent-street.

☞ *These splendid Plates ought to be in the library of every accoucheur.*

5. The Liverpool Medical Gazette, or Monthly Journal of Medicine and the Collateral Sciences, No. I. Jan. 1833, pp. 48, price 1*s*. Churchill, Princes-st. London.

6. On the Structure of the Human Placenta, and its Connexions with the Uterus, &c. By THOMAS RADFORD, Senior Surgeon to the Lying-in Hospital, &c. Manchester. Octavo, pp. 23, with three Plates. Manchester, 1832.

☞ *This investigation is very creditable to the author. He differs from some eminent physiologists of the day; but adduces good reasons for his opinions.*

7. Illustrations of the Elementary Forms of Disease. By ROBT. CARSWELL, M.D. Professor of Pathological Anatomy in the University of London. Fasciculus first—Tubercle. Folio, pp. 16, and four Plates, including numerous Figures, 1833.

8. Facts and Observations relative to the Disease commonly called Cholera, as it has recently prevailed in the City of York. By J. P. NEEDHAM, M.R.C.L. Octavo, pp. 138, 1833.

9. A further Examination of the Principles of the Treatment of Gout; with

Observations on the Use and Abuse of Colchicum. By Sir CHAS. SCUDAMORE, M.D. &c. Octavo, pp. 127. Second Ed. enlarged. 1833.

---

10. The Cholera Spasmodica, as observed in Paris in 1832, comprising its Symptoms, Pathology, and Treatment. By ABEL SMITH, M.D. Octavo, pp. 80. New York, 1833.

---

11. A Theoretical and Practical Treatise upon the Ligature of Arteries. Translated from the French of P. J. MANEC, by Messrs. GARLICK and COPPERTHWAITE, with notes and Appendices, selected from the Writings of many celebrated Surgeons. Quarto, pp. 227, with numerous coloured Plates, price £1. 17*s.* 6*d.* bds. Highley, London, Jan. 1833.

☞ *We have reviewed the original work in our last Number (p. 67—79), and have now only to recommend an excellent translation, with many valuable notes to our readers.*

---

12. Cyclopædia of Practical Medicine, Part XIV. Feb. 1833.

☞ *This Part contains the articles,* JAUNDICE—KIDNEY DISEASES—LACTATION—LARYNGITIS—LEPRA—LEUCORRHŒA—LICHEN—HEPATITIS—MALARIA—MALFORMATION OF HEART—PRACTICAL MEDICINE—MELENA—MENORRHAGIA—*and* MENSTRUATION. *The chief writers are* Drs. LOCOCK, CHEYNE, STOKES, BROWN, CONOLLY, *and* CARSWELL. *This Part maintains its reputation.*

---

13. Principles and Illustrations of Morbid Anatomy, adapted to the Elements of M. Andral and to the Cyclopædia of Practical Medicine, being a complete Series of coloured Lithographic Drawings, from Originals by the Author, with Descriptions, &c. By J. HOPE, M.D. Physician to the St. Marylebone Infirmary. Part II. Feb. 1833.

---

14. An Essay on Hydrophobia. By J. L. BARDSLEY, M.D. Octavo, pp. 146. 1832.

An Essay on Diabetes. By JAMES L. BARDSLEY, M.D. &c. Octavo, pp. . 1832.

☞ *Both these Essays are re-printed from the Cyclopædia of Practical Medicine, in which work they stand as able and conspicuous articles under their alphabetical heads.*

---

15. New Views of the Process of Defecation, and their Application to the Pathology and Treatment of Diseases of the Stomach, Bowels, and other Organs; together with an Analytical Correction of Sir Charles Bell's Views respecting the Nerves of the Face. By JAMES O'BEIRNE, M.D. Surgeon Extraordinary to the King, &c. Octavo, pp. 286. Dublin and London, Feb. 1833.

---

16. Memoir of the Life and Medical Opinions of John Armstrong, M.D. To which is added, an Inquiry into the Facts connected with those Forms of Fever attributed to Malaria or Marsh Effluvium. By FRANCIS BOOTT, M.D. Secretary to the Linnæan Society, &c. First Vol. 8vo. pp. 612. Feb. 1833; with a Profile and Autograph of Dr. Armstrong.

---

17. Principles of Obstetric Medicine, in a Series of Systematic Dissertations on Midwifery, &c. By DAVID D. DAVIS, M.D. Parts 14, 15, and 16, 1833, with Plates, price 2*s.* each.

---

18. A Demonstration of the Nerves of the Human Body, consisting of four Parts:—*Part the First,* the Cervical and Thoracic Portions of the Sympathetic, and the Nerves of the Thoracic Viscera. *Part the Second,* the Lumbar and Sacral Portions of the Sympathetic, and the Nerves of the Abdominal Viscera. *Part Three,* the Cerebral Nerves. *Part Four,* the Spinal Nerves. By JOSEPH SWAN. Part the Third, fourteen Pages of Letter-press and fourteen Plates, £3. 13*s.* 6*d.* Longman's Feb. 1833.

☞ *Such a magnificent and national work deserves the patronage of all public bodies, and of every private practitioner who has the means of encouraging merit.*

---

19. Remarks submitted to the Medical Professors of the Universities of Scotland and Ireland, as well as to the Licentiates, on the Necessity of Medical Reform, &c. By MACHAON. Octavo, pp. 16. Burgess and Hill, Feb. 1833.

☞ *We have touched on the same subject in the present Number.*

20. Researches on the Pathology and Treatment of some of the most important Diseases of Women. By Robert Lee, M.D. F.R.S. Physician-Accoucheur to the British Lying-in Hospital, &c. Octavo, pp. 220, with Plates, price 7*s*. 6*d*. Highley, Feb. 1833.

---

21. The Hunterian Oration for 1833. Mr. Howship, Octavo, pp. 28.

☞ *We were deprived of the pleasure of hearing this Oration. We are informed that Mr. Howship delivered it without looking at the MS. If so, he has more than the memory of a Porson and a Parr united. It is almost entirely historical, and traces medicine and surgery from the earliest records to the present time. It is a very creditable oration.*

---

22. The Outlines of Human Physiology. By Herbert Mayo, F.R.S. Professor of Anatomy in King's College, London. *The Third Edition.* Octavo. pp. 478, with Woodcuts and Engravings. March, 1833. Burgess and Hill, 18*s*.

☞ *See Periscope.*

---

23. Clinical Illustrations of the more important Diseases of Bengal, with the Result of an Inquiry into their Pathology and Treatment. By W. Twining, M.R.C.S. and assistant surgeon of the General Hospital of Calcutta. Octavo, pp. 705. Parbury and Allen. 1833.

☞ *This work should be in the hands of every medical man who sails for our Eastern possessions. We shall notice its contents in our next.*

---

24. A Discourse delivered on commencing the Lectures in Jefferson Medical College. By Granville Sharp Pattison, M.D. &c. Philadelphia, 1832.

---

25. Letter on Cholera from Professor Pattison to Dr. Carmichael, of Virginia. Philadelphia, 1832.

☞ *We are gratified to see that our distinguished countryman has ably and eloquently advocated the non-contagious nature of the late epidemic in the United States. An overwhelming majority of the most talented physicians in America are of opinion that the disease was* not *imported into the United States, but originated there from causes in the earth or atmosphere, in the same way as other epidemics.*

---

26. A Discourse introductory to the Course of Lectures on the Theory and Practice of Physic, in the Jefferson Medical College, 1832-3. By J. Revere, M.D.

☞ *A very appropriate discourse.*

---

27. Anatomie Pathologique du Corps Humain, &c. Par J. Cruveilhier, Professeur d'Anatomie, &c. Thirteenth, fourteenth, and fifteenth Livraison. Bailliere, 1830-31.

---

28. A Treatise on Indigestion and its Consequences, called Nervous and Bilious Complaints, with Observations on the Organic Diseases in which they sometimes terminate. By A. P. W. Philip, M.D. F.R.S. Seventh Edition, improved, Renshaw and Rush, March, 1833.

---

29. The Dublin Journal of Medical and Chemical Science, No. VII. March, 1833.

☞ *This contemporary is rapidly acquiring strength, and consequent circulation*—MOBILITATE VIGET.

---

30. Letters on Cholera Asphyxia, as it has appeared in the City of New York. By Martin Payne, M.D.

☞ *The mercurial treatment appears to have been the most successful in New York. The idea of importation and contagion, in respect to cholera, is scouted by all the most scientific observers in America.*

---

31. The Dissector. By R. Dewey Forster. Part the First.

The Demonstrator; being an Explanation of the Dissector, in 16 Parts, Part the First, 8vo.

☞ *This ingenious work will be noticed in our next.*

---

32. Traité Pratique des Maladies de l'Uterus et de ses Annexes, &c. Par Mad. Boivin et par A. M. Duges. Fifth Fasciculus. Bailliere, March, 1833.

☞ *This work will be found analyzed in this Journal regularly.*

### INDEX.

A.

Abouzabel, Clot Bey's school at.... 545
Abscess of the tibia, Mr. Brodie on 33
Abscess in the heart, case of ...... 174
Abscess in lung, opening externally 182
Abscess of the prostate .......... 445
Abscess of the pelvis after lithotomy 571
Abscess of the pelvis, cases of...... 572
Absorption by skin, experiments on 22
Academy of Medicine, France, proceedings at the ............... 543
Acephalism, case of.............. 209
Acephalocystes, remarks on ...... 544
Actual cautery in vesico-vaginal fistula......................... 232
Adams, Mr. on congenital hernia .. 486
Air, alterations of, from respiration 26
Air in the circulating organs, accidents from.................... 517
Amaurosis from contusion ........ 579
Amphibious Animals—M. Buffon's idea......................... 18
Amputation of penis, case of ...... 223
Anatomical plates, Mr. Schloss's .. 261
Anatomical atlas of Weber........ 283
Anatomy, Mr. B.Cooper's lectures on 97
Anatomy, Mr. Quain's elements of.. 242
Aneurism, ligature of subclavian for 247
Aneurism of gluteal artery........ 476
Aneurism by anastomosis, cases of, 505
Aneurisms, false, of brachial artery 315
Angina pectoris, Dr. Copland on, in Dict. ....................... 112
Animal heat, uniformity of........ 23
Animal electricity and muscular contractions .................... 211
Animals, young, temperature of .. 10
Antwerp, surgical account of siege of 578
Anus, fissure of, cured by belladonna 187
Aphonia, Dr. Webster on ........ 551
Aphorism of Galen .............. 514
Argenti nitras, mode of applying on a probe ....................... 187
Armstrong, Dr. life of............ 374
Armstrong, Dr. his start in London 376
Armstrong, Dr. his rejection ...... 378
Armstrong, Dr. his malady ...... 379
Armstrong, Dr. his post-mortem examination .................... 382
Armstrong, Dr. his character...... 383
Arteries, M. Manec's treatise on .. 67
Arteries, anatomy of ............ 68
Arteries, effects of ligatures on .... 70
Arteries, effect of small round ligature 72
Arteries, effect of large flat ligature 74
Arteries, torsion of .............. 75
Arteries, ossified ................ 77
Arteries, wounds of.............. 77
Arteries, Dr. Smith's anatomy of the 255
Articulation, Sir C. Bell on......... 556
Arts, trades, &c. Mr. Thackrah on 101
Asphyxia, Dr. Edwards on ........ 17
Atmosphere, influence of, on batrachian reptiles ................ 2

B.

Ballingall, Dr. on military surgery.. 428
Bell, Sir C. on the human voice.... 552
Bennati, M. on the mechanism of the voice ....................... 79
Bladder, Dr. Macfarlane on wounds of the ....................... 443
Bladder, inversion of the.......... 469
Blennorrhagia, acute, M. Ricord on 389
Blennorrhagia, chronic, M. Ricord on ......................... 390
Blood, composition of, in jaundice.. 490
Blood, pathology of, in cholera .... 515
Bloomfield, Mr. on abscess of tibia 36
Blue urine ..................... 541
Boivin, Madame, on diseases of the uterus......................... 406
Boott, Dr. his life of Dr. Armstrong.. 374
Brain, Mr. Crampton on inflammation of....................... 237
Brass-founders, diseases of........ 103
Brigham, Mr. his case of empyema 450
Brodie, Mr. on abscess of the tibia 33
Bronchotomy, Mr. Wood on ...... 345
Brookes, miniature bust of......... 581
Broussais's doctrines, sketch of.... 469
Bryce, Dr. on cholera ............ 195
Buchanan's history of the Glasgow infirmary ................ ... 559

C.

Calculi removed by Weiss' dilator.. 224
Calculus in the bladder, cases of .. 127
Calculus extracted by Weiss' forceps 547
Calf of leg, malignant tumor in.... 279
Cameron, Mr. on variety in diet.... 94
Camps and garrisons, diseases of .. 429
Cancer of stomach, without symptoms....................... 467

Carmichael, Mr. on tracheotomy .. 227
Carotid, tied for hemiplegia ...... 253
Carswell, Dr. on morbid anatomy.. 366
Cataract, M. Dupuytren on ...... 295
Catheter, death from introduction of 249
Cerebral obstruction, Mr. Howship on ........................ 44
Chick, on the evolution of the .... 173
Cholera, epidemic, Mr. Corbyn on 63
Cholera, Mr. Corbyn's treatment of 64
Cholera, previous to 1816 ........ 66
Cholera at Dantzick, Dr. Hamett on 136
Cholera, Dr. M'Cormack on ...... 152
Cholera simulated by gastro-enterite 173
Cholera, different modes of treatment of, in Paris .............. 185
Cholera anecdotes................ 186
Cholera, occurrences antecedent to its outbreak ................ 186
Cholera, etiology of, in Glasgow.... 195
Cholera, Mr. Watt on ............ 198
Cholera, proximate cause of ...... 199
Cholera in Scotland .............. 216
Cholera, what is its contagion ? .... 245
Cholera, Dr. Haslewood and Mr. Mordey on.................... 261
Cholera, Dr. Keir on ............ 262
Cholera, Mr. Morrah on Cupri Sulphas in ...................... 263
Cholera, Dr. Tweedie on ........ 263
Cholera, Mr. Steward on .......... 264
Cholera, Dr. Ayre on ............ 264
Cholera, Mr. Hickman on ........ 264
Cholera, Messrs. Tweedie and Gazelee on ...................... 265
Cholera, letter on................ 268
Cholera-phobia in Ireland ........ 268
Cholera, Dr. Thomson on ........ 269
Cholera, charcoal in.............. 272
Cholera, Dr. Williams on ........ 272
Cholera, cutaneous eruption in .... 466
Cholera and sweating sickness at Pontoise ...................... 514
Cholera, pathology of the blood in, 515
Circulating organs, on the presence of air in ...................... 517
Clavicle, case of dislocation of .... 165
Clerkships and house-surgencies, should be gratuitous .......... 562
Clinical reports, Dr. Macfarlane's.. 126
Clinical remarks on opium by Dr. Stokes ...................... 156
Clinical instruction, hints on...... 226
Clinical lectures of Dupuytren .... 289
Clinical reporting, remarks on .... 560
Clinical lectures, remarks on...... 561
Clinique of Dupuytren .......... 530
Clinique of M. Bouillaud.......... 533
Clitoris and nymphæ, morbid enlargements of ................ 448
Clot Bey on the Guinea-worm .... 483
Clot Bey on elephantiasis ........ 529
Clot Bey's School of Medicine at Abouzabel .................... 545
Cold, Dr. Clendinning on ........ 200
Cold, prevention of diseases from .. 202
Cold affusion in cerebral cases .... 205
Cold affusion in tetanus .......... 526
College of physicians; reform .... 582
Compound fracture of the femur, case of........................ 162
Compound fracture of cranium, trephining in.................... 509
Congenital hernia, Mr. Adams on.. 486
Congenital enterocele ............ 486
Congenital encephalocele ........ 487
Contagion, fear of................ 148
Contagion of diseases ............ 187
Contagionists, veracity of ........ 271
Contusions from gunshot ........ 579
Cooper, Mr. B. on anatomy........ 97
Copland, Dr. his dictionary of medicine........................ 111
Copper-plate printers, diseases of.. 104
Copper, sugar an antidote to ...... 528
Cord, inflamed, mistaken for hernia, 468
Counteraction as a means of cure .. 170
Crampton, Mr. on inflammation of brain, &c...................... 237
Cyanuret of potassium in neuralgia, 525
Cyclopædia of practical medicine .. 362
Cyst in the orbit, containing hydatids 342
Cystocele, vaginal, case of ........ 208

D.

Davis, Dr. his obstetric medicine .. 491
Death from lithotomy, cases and remarks.......................... 564
Delpech, M. assassination of ...... 519
Diabetes mellitus, case of ........ 176
Diabetes mellitus, case of ........ 192
Dictionary of practical medicine, Dr. Copland's .................... 111
Diet, Mr. Cameron on............ 94
Diffuse inflammation after bleeding in foot........................ 462
Diffuse cellular inflammation after lithotomy .................... 565
Disease transmitted along an electric wire. .................... 215
Dislocation of the clavicle ........ 165
Dislocation of the humerus ...... 165
Doctors deceived ................ 231
Dothinenterite, cases of.......... 193
Drowning, Sir Astley Cooper's experiments on.................. 19
Dublin fever hospital, report of.... 149
Dugès, M. on diseases of the uterus 406
Dumfries meeting on cholera...... 216
Dupuytren on cataract .......... 295

Dupuytren on permanent retraction of fingers .................... 290
Dupuytren on enlargements of the testis ...................... 300
Dupuytren on traumatic emphysema 302
Dupuytren on fistulous sinuses .... 303
Dupuytren on prolapsus recti...... 305
Dupuytren on traumatic delirium.. 306
Dupuytren on fractures of fibula .. 308
Dupuytren on false aneurism of brachial artery .................. 315
Dupuytren on fractures of patella .. 316
Dupuytren on fractures in general 317
Dupuytren on excision of hæmorrhoids ......................... 318
Dupuytren's clinical lectures, review of ...................... 289

E.

Edwards, Dr. on the influence of physical agents................ 1
Electric wire, transmissibility of diseases along .................. 215
Elephantiasis, Clot Bey on ...... .. 529
Emphysema, pulmonary, in sudden death ......................... 516
Empyema ; successful operation .. 450
Encephalon, memoir on the functions of the .................... 522
Endermic therapeutics ........... 176
Endosmosis and exosmosis ........ 213
Epidemic cholera, Mr. Corbyn on.. 63
Epidemic influenza ............... 154
Epidemics, Baron Alibert on the causes of...................... 477
Epidemics, march of ............. 500
Epidemics, independent of atmospheric changes ................ 535
Epilepsy, carotid tied for.......... 254
Epps, Dr. on counteraction........ 170
Erysipelas treated by mercurial inunction ...................... 175
Evolution of the chick, Carus on the 171
Excerpta choleralogica ........... 261
Excision of head of humerus, cases of 274
Exostoses, large, arising in infancy, 167

F.

Fear of contagion................ 148
Females, venereal disease in ...... 388
Fever, report on, by Dr. Hackett.. 149
Fever, after operations .......... 404
Fingers, state of, in phthisis ...... 189
Fingers, on permanent retraction of 290
Fingers, permanent flexions of the, 461
Fishes, as affected by physical agents 8
Fishes, life of, out of water ........ 9
Fistula ani, Mr. Liston's treatment of 330
Fistulous sinuses, M. Dupuytren on 303
Fracture, compound of the femur .. 162
Fractures of the fibula, M. Dupuytren on ........................ 308
Fractures of the patella, M. Dupuytren on ...................... 316
Fractures of the extremities, M. Dupuytren on.................... 317
Fractures of bones by muscles .... 531
Frogs, in water, existence of ...... 4

G.

Gangrene, spontaneous, case of.... 182
Gastro-enterite, simulating cholera, 173
Gilders, diseases of .............. 105
Gilkrest, Dr. on yellow fever...... 362
Glasgow Infirmary, Dr. Buchanan's history ....................... 559
Glossitis, Cyclopædia article on .... 234
Gluteal aneurysm................. 476
Gold and silver, workers in, diseases of ....................... 107
Goitre in the plains of the Andes, Humboldt on ................. 520
Gravel, M. Majendie on ........... 49
Gravel, formation of ............ 50
Gravel, red .................... 51
Gravel, red, causes of ............ 52
Gravel, white .................... 53
Gravel, hairy..................... 54
Gravel, grey ..................... 54
Gravel, yellow ................... 54
Gravel, transparent .............. 55
Gravel, peculiar causes of ....... 55
Gravel, symptoms of.............. 56
Gravel, treatment of ............. 56
Graves, Dr. on periostitis ........ 481
Groin, cases of obstinate ulceration in the ....................... 282
Guinea worm, Clot Bey on the .... 483

H.

Hackan, Dr. his report on fever.... 149
Hæmorrhage, experiments relating to............................ 256
Hall, Dr. on loss of blood ........ 354
Hamett, Dr. on cholera at Dantzick 136
Hancock, Dr. on external stimulants 145
Hawkins, Mr. on the arrest of hæmorrhage ..................... 38
Hawkins, Mr. experiments of...... 39
Hawkins, Mr. conclusions of ...... 41
Head, injuries of the ............ 506
Hearing, Dr. Caswall on.......... 537
Heart, abscess in the ............ 174
Heart, case of wound of .......... 453
Heart, new theory of sounds of .... 454
Heart; M. Pigeaux's theory ...... 455
Heart, spontaneous ruptures of.... 455
Heart, malformation of ........... 456
Heart, Dr. Billing's theory of its sounds ......................... 564

Hemiplegia, carotid tied for ...... 253
Hernia, without external swelling.. 463
Hernia, cord inflamed, mistaken for 468
Hewlett, Mr. his case of ovarian disease....................... 46
Hibernation, Dr. Edwards on ..... 13
Hibernation, facts relating to...... 15
Hooping-cough, Dr. Autenrieth's treatment of .................. 527
Hope, Dr. on morbid anatomy .... 284
Hope, Dr. on morbid anatomy .... 367
Hospitals, military, history of .... 431
Howship, Mr. his case of cerebral disease ....................... 44
Humboldt, M. on goitre in the Andes 520
Humerus, dislocation of, into the axilla ........................ 165
Humerus, excised and amputated; cases ...................... 274
Husbandmen, healthfulness of .... 102
Hybernation, Dr. M. Hall on...... 497
Hybernation, respiration during.... 498
Hybernation, irritability, &c. during 499
Hydatid cysts in abdomen and thorax 514
Hydrocele, spontaneous cure of.... 489
Hydrocephalus, spurious, with psoas abscess ...................... 160

I.

Ilium, enormous tumor attached to, 236
Inflammation of the brain, Mr. Crampton on ................ 237
Inflammations, secondary, M. Velpeau on ...................... 398
Inflammations, secondary, history of 399
Inflammations, secondary, treatment 405
Influenza, epidemic.............. 154
Insanity, Dr. Seymour on ........ 139
Insanity, treatment of............ 141
Insanity; post-mortem appearances, 502
Insanity in France ................ 531
Institute of France, proceedings at, 540
Intestines, Dr. Wise on wounds of.. 250
Inversio vesicæ, case of .......... 459
Irritability and respiration, connexion between ................. 2
Issues, on the employment of...... 512

J

Jaundice, composition of blood in .. 490
Jelly from bones ........... .... 543

K.

Kidneys, cases of disease in the.... 190

L.

Lacerated arteries, cessation of hæmorrhage from................ 256
Lacerated arteries, experiments on 257
Lacerated perinæum, cured by suture 526
Language, singular affection of organ of ...................... 539
Laryngeal fistula, cured .......... 194
Larynx, mercury in chronic affections of ...................... 231
Larynx, Mr. Wood on inflammation of ......................... 343
Lawrence, Mr. on tumours........ 333
Lead, white, workers in; diseases of 109
Life, value of, in males and females 493
Light, influence of, on animal bodies 26
Liston, Mr. his elements of surgery 328
Liston, Mr. on lithotomy & lithotrity 331
Lithotomy, Dr. Macfarlane on .... 127
Lithotomy, death from............. 564
Loss of blood, Dr. M. Hall on...... 354
Lung, case of abscess in the ...... 182
Lymphatics, anatomy and use of .. 214

M.

M'Cormac, Dr. on cholera ........ 152
Macfarlane, Dr. his clinical reports 126
Macfarlane, Dr. on aneurysm, &c.. 505
Magnetism and electricity, identity of 458
Majendie, M. on gravel .......... 49
Males and females, value of life in.. 493
Malignant tumour in calf of leg, cases of ...................... 279
Manec, M. on ligature of arteries.. 67
Materia medica, Dr. Thompson on.. 321
Med.-Chir. Transactions, Vol. VII... 33
Mémoires de l'Academie Royale de Médecine .................... 387
Memory, loss of ................ 548
Mercury in chronic affections of the larynx....................... 231
Middlemore, Mr. on purulent ophthalmia ...................... 511
Miliary fever at Auxi-le-Chateau .. 521
Military surgery, Dr. Ballingall's lectures on................... 428
Milk, a remedy for dropsy ........ 541
Moles, varieties of .............. 421
Moon, influence of, on menstruation 491
Morbid anatomy, Dr. Hope's...... 284
Morbid anatomy, works on........ 366
Morbid anatomy, Dr. Carswell on.. 366
Morbid anatomy, Dr. Hope on .... 371
Morbus cæruleus, curious case of .. 516
Morgan, Mr. on tetanus.......... 536
Mortality of Paris in 1830 ........ 181
Muscular contractions, Dr. Edwards on ......................... 211

N.

Nautilus, pearly, Mr. Owen on the 123
Nephritis, acute, case of.......... 190
Nerves Mr. Swan's splendid work on the ........................ 385
Neuralgia, cyanuret of potassium in 525

O.
Obstetric medicine, Dr. Davis's.... 491
Operations, some rules respecting .. 396
Operative surgery, M. Velpeau's .. 395
Opium, Dr. Stokes on large doses of 156
Osteo-sarcoma of the arm ........ 166
Ovarian disease complicated with pregnancy .................... 46

P.
Paillard's account of the siege of Antwerp.................... 578
Paris, Bill of Mortality in, for 1830.. 181
Patella, Sir C. Bell on fracture of.. 482
Pearly nautilus, Mr. Owen on the.. 123
Pellagra, notice of .............. 176
Pellagra, cases of................ 178
Penis, injury and amputation of.... 223
Pericarditis, case of .............. 457
Pericarditis, case of.............. 464
Pericardium, universal adhesions of 501
Periostitis, Dr. Graves on ........ 481
Perspiration, Dr. Edwards on...... 21
Phthisis, state of fingers in........ 189
Physic and surgery, their distinctions 225
Physical agents, Dr. Edwards on .. 1
Physiological doctrine, Broussais'; sketch of .................... 469
Pneumonia, tartar-emetic in ...... 175
Pneumonia, tartar-emetic in ...... 479
Poisoning, case of, by Foderé...... 183
Poisoning by sulphuric acid........ 530
Portal, M. death of .............. 475
Post-mortem appearances in insanity 502
Prizes of the Institute of France .. 542
Prolapsus of rectum, M. Dupuytren on .......................... 305
Prolapsus ani, Dr. Macfarlane on .. 447
Prostate gland, abscess of ........ 445
Psoas abscess, connected with hydrocephalus .................. 160
Pulmonary and cutaneous functions in frogs........................ 6
Purulent deposites and phlebitis .. 398
Purulent ophthalmia, Mr. Middlemore on...................... 511
Purulent deposites, theories of .... 402

Q.
Quain, Mr. his elements of anatomy 242

R.
Radix caincæ, as a diuretic ...... 501
Re-action, Dr Hall on............ 355
Recollection and memory; distinctions ...................... 549
Rectum, on inflammation of the .. 329
Reform, College of Physicians .... 582
Rete mucosum, remarks on the.... 352
Revulsion, Sabatier on the laws of.. 87
Revulsion, cases of .............. 89
Revulsion, applicability of ........ 91
Revulsives, two kinds of .......... 88
Rhinoplastic operation, case of .... 188
Ricord, M. on the venereal........ 388
Rolando, M. on the functions of the encephalon .................. 522
Ruptures, spontaneous, of heart.... 455

S.
Sabatier on the laws of revulsion .. 87
St. George's Hospital report ...... 564
St. Vitus' dance, treatment of, in France ...................... 466
Seasons, effects of, on animals .... 17
Seiler, Dr. on the uterus and ovum 356
Serous cysts, Mr. Lawrence on .. .. 342
Seymour, Dr. on insanity ........ 139
Shoulder-joint, excision and amputation at.................... 274
Skin, Dr. Wood on the .......... 350
Smoking, is it pernicious? ........ 489
Speculum, facts relating to the use of 389
Spurzheim, Dr. death of.......... 532
Stiff knee, recovery from, in fever.. 468
Stimulants, external, Dr. Hancock on 145
Stokes, Dr. on large doses of opium 156
Stomach, organic disease of ...... 469
Stomach, ulceration of the........ 477
Stump, French mode of dressing .. 176
Styptics, Mr. Hawkins on ........ 38
Subcarbonate of iron in gastralgia.. 530
Subclavian artery, ligature of the .. 247
Sugar an antidote to copper ...... 528
Surgery, Liston's elements of...... 328
Syncope, Dr. Hall on ............ 355
Syphilis, treated by mercury externally ...................... 194
Syphilis, cyanuret of mercury in .. 527
Swan, Mr. on the cerebral nerves .. 385
Sweating fever at Auxi-le-Chateau.. 521

T.
Tadpoles, Dr. Edwards on the development of.................. 7
Tartar-emetic in pneumonia ...... 479
Temperature of young animals .... 10
Temperature of adult, warm-blooded animals .................... 12
Temperature, high, effects of...... 110
Tepid baths, with cold affusions.... 205
Testis, M. Dupuytren on enlargements of.................... 300
Tetanus, cold affusion in ........ 526
Tetanus, Mr. Morgan's lecture on.. 536
Thackrah, Mr. on arts, trades, &c.. 101
Thompson, Dr. on materia medica.. 321
Thompson, Dr. his classification .. 327
Throat, Mr. Wood on wounds of .. 347
Tibia, cases of abscess of the...... 33

Toad-fish of Van Dieman's Land .. 273
Tobacco, bad effects of smoking .. 489
Tongue, case of cancer of the...... 194
Trachea, foreign body in, 5 months 535
Tracheotomy, Mr. Carmichael on .. 227
Traité des maladies de l'uterus .... 406
"Trances," remarks on .......... 28
Traumatic emphysema, M. Dupuytren on .......................... 303
Traumatic delirium, M. Dupuytren on .......................... 306
Tubercle, Dr. Carswell on ........ 367
Tubercle, Dr. Hope on............ 371
Tumour, enormous, attached to ilium 236
Tumours, Mr. Lawrence on ...... 333
Tumours, cellular................ 335
Tumours, in the vicinity of parotid 336
Tumours diagnosis of ............ 339

U.

Ulceration in the groin, obstinate and fatal...................... 282
Ureter, obliteration of the ........ 192
Urethra, contusions of the ........ 443
Urinary abscess, case of .......... 444
Uterus and human ovum, Dr. Seiler on ....... .................. 358
Uterus, M. Dugés and Mad. Boivin on diseases of ................ 406
Uterus, anteversion of the ........ 407
Uterus, hernia of the ............ 408
Uterus, unnatural fixedness of the.. 410
Uterus, flexion of the ............ 412
Uterus, reversion of os tincæ of.... 413
Uterus, adhesions, &c. of os tincæ of 413
Uterus, incurvations of .......... 414
Uterus, retroflexion of; cases...... 417
Uterus, distention of, by gas ...... 418
Uterus, distention of, by water.... 419
Uterus, calculi of ................ 420
Uterus; moles .................. 421
Uterus, degenerations of.......... 425
Uterus, vascular degeneration ...... 425
Uterus, cellular excrescence of .... 425
Uterus, fibrous and cartilaginous degenerations ................ 426
Uterus; tubercular degeneration .. 427

V.

Vagina, imperforate, case of ...... 449
Vaginal cystocele, case of ........ 208
Van Dieman's Land, toad-fish of .. 273
Vapour and warm-baths .......... 29
Variety in diet, Mr. Cameron on .. 94
Variolous epidemic, at Turin...... 529
Velpeau, M. his operative surgery, 395
Venereal disease, M. Ricord on the, 388
Vesico-vaginal fistula, actual cautery in ............................ 232
Voice, M. Bennati on the.......... 79
Voice affected by the velum palati.. 80
Voice in many eminent singers.... 82
Voice, influence of the larynx .... 83
Voice, Sir C. Bell on the .......... 552
Voice; influence of the trachea and larynx........................ 553

W.

Water, as an external agent ...... 476
Weiss' dilator, calculi removed by.. 224
Weiss' forceps, calculus extracted by 547
Wood, Mr. on inflammation of the larynx........................ 343
Wood, Mr. on wounds of the throat 247
Wood, Dr. on the skin............ 350
Wound of heart, case of .......... 453
Wounds of intestines, Dr. Wise on, 250
Wounds of the bladder, Dr. Macfarlane on ...................... 443

Y.

Yellow fever, Dr. Gilkrest on...... 362
Yellow fever, treatment of........ 363

---

MEDICO-CHIRURGICAL REVIEW, No. XXVII.

THE Editor would feel under particular obligations to any gentleman holding the above Number of the Journal (and not having a complete set) to return it to the Publisher (Mr. Highley) who will pay the full price for it. The subject of Cholera has caused the above Number to be prematurely exhausted, and consequently prevents the completion of sets of the work.

*** Several Works for Review came to hand after the Bibliographical Record was worked off.

